# Long-term safety of Mometasone Furoate administered via a dry powder inhaler in children: Results of an open-label study comparing Mometasone Furoate with Beclomethasone Dipropionate in children with persistent asthma

**DOI:** 10.1186/1471-2431-9-43

**Published:** 2009-07-13

**Authors:** Michael Noonan, Jeffrey Leflein, Jonathan Corren, Heribert Staudinger

**Affiliations:** 1 Allergy Associates Research Center, LLC, Portland, OR USA; 2 Allergy & Immunology Associates, Ann Arbor, MI, USA; 3 Allergy Research Foundation, Inc., Los Angeles, CA, USA; 4 Schering-Plough Corp., Kenilworth, NJ, USA

## Abstract

**Background:**

To assess the long-term pediatric safety of 2 doses of mometasone furoate administered via a dry powder inhaler (MF-DPI) for mild-to-moderate persistent asthma and compare them with that of beclomethasone dipropionate administered via a metered dose inhaler (BDP-MDI) in the treatment of persistent asthma. Both MF-DPI doses tested are twice the approved pediatric dosage of 100 μg once-daily (QD) for children aged 4–11 years.

**Methods:**

Children (N = 233) aged 4–11 years were randomized to 52 weeks of treatment with MF-DPI 200 μg QD AM, MF-DPI 100 μg twice daily (BID), or BDP-MDI 168 μg BID. Patients had used inhaled corticosteroids (ICSs) daily for ≥ 30 days before the screening visit and were on stable ICS doses for ≥ 2 weeks before screening. The primary safety variable was the incidence of adverse events. Secondary safety variables were laboratory tests (including cortisol concentrations), vital signs, and physical examination.

**Results:**

The incidence of adverse events was similar in all 3 treatment groups. The most frequently reported adverse event was upper respiratory tract infection, reported by 47%–49% of the MF-DPI-treated patients and 51% of the BPD-treated patients. Most adverse events were considered unrelated to study drug. The most frequently reported related adverse events were headache (MF-DPI 200 μg QD AM, 8%; MF-DPI 100 μg BID, 4%; BDP-MDI 168 μg BID, 2%) and oral candidiasis (4% in each treatment group). No clinically relevant changes in laboratory values, including plasma cortisol, vital signs, or physical examinations were noted in any treatment group.

**Conclusion:**

Both MF-DPI doses were well tolerated, with no unusual or unexpected adverse events or safety concerns, and had a similar adverse event profile to that of BDP-MDI 168 μg BID.

## Background

Inhaled corticosteroids (ICSs) are considered to be the most effective controller medication for children with asthma.[1,2] However, safety concerns include their transient retardation of growth velocity and potential for hypothalamic-pituitary-adrenal (HPA)-axis suppression. The possibility of HPA-axis suppression is one of the more important issues with the long-term use of ICS therapy when high doses are used.

Mometasone furoate administered via a dry powder inhaler (MF-DPI) is an ICS indicated for the maintenance treatment of asthma in adults and children ≥ 4 years of age. MF-DPI is available commercially as Twisthaler^®^. The device has several features that make it attractive for use in children. It is easy to use and delivers uniform doses across inspiratory flow rates as low as 28.3 L/min.[3,4]

Three clinical trials in asthmatic adults have confirmed that at therapeutic doses MF-DPI does not have clinically meaningful effects on HPA-axis function as measured by serum cortisol, which has been shown to be an accurate measure of HPA-axis function. [5-7]

This study was designed to evaluate the long-term safety of MF-DPI 200 μg once daily in the morning (QD AM) and MF-DPI 100 μg twice daily (BID) against the safety characteristics of beclomethasone dipropionate administered via a metered dose inhaler (BDP-MDI) in children with asthma requiring ICS therapy. The doses tested in this clinical trial were twice the approved dose of 100 μg QD PM for children ages 4–11.[8] The safety variables evaluated over 1 year of treatment were adverse events (AEs), vital signs, physical examination, and laboratory tests (including analysis of plasma and urinary cortisol concentrations).

## Methods

The study was conducted in compliance with the human experimentation guidelines of the US Department of Health and Human Services and the Helsinki Declaration, 1975, last amended in Edinburgh, Scotland, 2000. The protocol and consent form were approved by an Institutional Review Board from each study center. Written informed consent was obtained from parents or legal guardians of each study participant.

### Patient Population and Demographics

This was an open-label, multicenter, prospective study. Children aged 4–11 years with a diagnosis of persistent asthma of at least 6-months' duration participated in the study. Eligible patients had a forced expiratory volume in 1 second (FEV_1_) of ≥ 60% of predicted at screening and baseline. In addition, patients must have been using ICSs for at least 30 days before screening, the regimen of which must have been stable for at least 2 weeks before screening. Patients must have demonstrated evidence of an increase in absolute FEV_1 _of ≥ 12% reversibility at screening or historically within the past 12 months and had plasma cortisol levels at screening (8 AM ± 1 h) of ≥ 5 μg/dL. Clinical laboratory tests (complete blood count, blood chemistry, urinalysis) were required to be within normal limits or clinically acceptable to the investigator. Eligible female enrollees were premenarchal. Patients were excluded if they had an upper- or lower-respiratory tract infection within 2 weeks of screening or baseline visits, demonstrated a ≥ 20% decrease in FEV_1 _between screening and baseline visits, or received prohibited medications including short-acting β-adrenergic inhaled bronchodilators within 6 hours, long-acting β_2_-agonist (LABA) inhaled bronchodilators within 1 week, and systemic corticosteroids within 1 month of screening visit. Also excluded were patients who required daily use of nebulized β-agonists or LABAs to control asthma, patients who had used >12 inhalations per day of albuterol on any 2 consecutive days between screening and baseline visits and patients who were unable to use the MF-DPI device or peak flow meter.

### Study Design

Data were collected in the United States and Puerto Rico between May 1998 and March 2000. After a 1- to 2-week run-in period on stable doses of previous therapy, patients were randomized to receive one of three treatments over a period of 52 weeks: MF-DPI 200 μg QD AM, MF-DPI 100 μg BID (both twice the FDA approved dose), or BDP 168 μg BID (FDA approved). Office visits occurred at weeks 1, 2, 4, 8, 12, 16, 26, 38, and 52. Telephone contacts occurred at weeks 20, 32, 42, and 48. An albuterol metered-dose inhaler was provided at the screening visit and enrollees were instructed to use it as needed, before exercise, and before exposure to potential triggers (eg, animals). Patients were also instructed to withhold albuterol use for at least 6 hours before each office visit. The use of any LABA was prohibited for the duration of the study.

### Study Medication

Patients received their first dose of study medication in the investigator's office. For patients assigned to BID treatment groups (MF-DPI 100 μg BID and BDP 168 μg BID), if the baseline visit was held before noon, the first dose of study medication (taken in the office) was to be considered the morning dose for that day; if the baseline visit was held after noon, the first dose of study medication (taken in the office) was to be considered the evening dose for that day. For patients in the MF-DPI 200 μg QD AM group, the first dose of study medication (taken in the office) was to be considered their daily dose, independent of the time of administration. Compliance was evaluated throughout the study by asking the patient and/or the parent/guardian whether all medications had been taken as instructed. The definition of noncompliance was the use of <75% or >125% of the specified dose.

### Safety Assessments

Safety was assessed by measuring the incidence and severity of AEs, vital signs, laboratory findings, and physical examination, including oropharyngeal and ophthalmic examination. Safety analyses were based on the intent-to-treat-population, defined as any patient who took at least 1 dose of study medication. The primary safety criteria were incidence and severity rates of AEs between groups. AEs were judged by investigators to be not related, possibly related, probably related, or related to therapy. Serious adverse events (SAEs) were defined as AEs that were fatal, life threatening, significantly or permanently disabling, or resulting in inpatient or prolonged hospitalization. Incidences of cancer, overdose (intentional or inadvertent), or congenital anomalies were also considered SAEs. Asthma worsening was defined as a ≥ 20% drop in FEV_1 _from baseline; a clinical asthma exacerbation that required hospitalization, treatment with asthma medication other than short-acting inhaled β-agonists, or any other emergency treatment for asthma; a ≥ 25% drop in AM or PM peak expiratory flow from the baseline AM PEF for 2 consecutive days; Proventil^® ^MDI use of ≥ 12 inhalations per day for 2 consecutive days; or >2 treatments with nebulized beta agonists on 2 consecutive days. Patients who experienced asthma worsening may not have discontinued study treatment. Patients were discontinued because of significant asthma worsening if they required oral steroid treatment for >15 days during the study if there was an interval of ≥ 4 weeks between the last dose of systemic steroid and 8 AM plasma cortisol and 12-hour urinary cortisol testing; hospitalization for asthma occurred more than twice; ventilator support was required; or chronic treatment with additional ICSs was required. Morning (8 AM) plasma cortisol and 12-hour urinary cortisol levels were measured at screening (plasma cortisol) or baseline (urinary cortisol), week 26, and week 52 to evaluate HPA axis function in patients at 10 preselected centers. MF-DPI plasma concentrations were measured using predose samples collected at weeks 26 and 52; concentrations <50 pg/mL were below the limit of quantitation. As this was primarily a long-term safety study, the study was not designed or powered to detect any treatment differences in efficacy variables.

### Statistical Analysis

Adverse events, vital signs, laboratory tests including 8 AM plasma cortisol and 12-hour urinary cortisol concentrations, and physical examination findings including oropharyngeal and ophthalmic exam results were summarized by treatment group. For vital signs and laboratory tests, endpoint was defined as the measurement taken closest to the last day of study treatment. Kaplan-Meier estimates of time to first asthma worsening were calculated using the treatment day of the first observation of an asthma worsening event and compared between treatment groups using a log-rank test. Patients who met the criteria for asthma worsening which required discontinuation were classified as having been discontinued for the purposes of this analysis, regardless of whether or not an investigator actually discontinued the patient.

## Results

### Demographics

**Figure 1:**
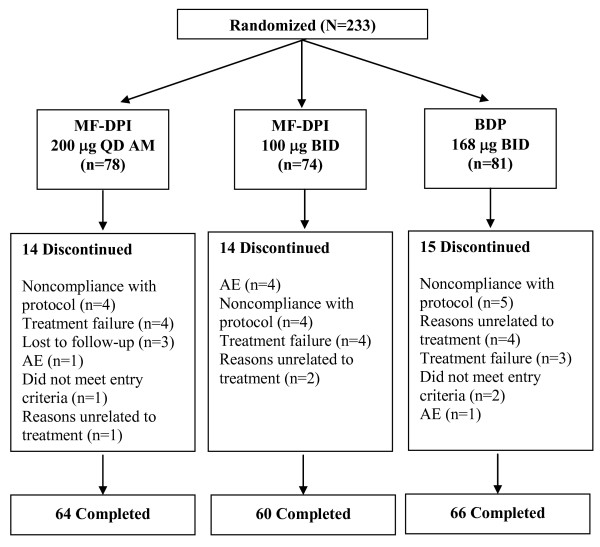
**Patient disposition**. AE = adverse event; BID = twice daily; BDP = beclomethasone dipropionate; MF-DPI = mometasone furoate administered via a dry powder inhaler; QD AM = once daily in the morning.

A total of 233 patients were enrolled and 190 patients completed the study (Figure 1). Among all patients who discontinued study treatment (n = 43), the most common reason for discontinuation was noncompliance with protocol (n = 13; Figure 1). Baseline and postbaseline data for plasma cortisol and 12-hour urinary cortisol were available for 88 and 87 patients, respectively. The mean age in each treatment group was approximately 8 years. The majority of patients was boys (60%) and white (80%). A detailed list of demographics and clinical characteristics of the studied population are shown in Table 1.

**Table 1:** Baseline Patient Demographics and Clinical Characteristics

**Demographic or Characteristic**	**MF-DPI** **200 μg QD AM** **(n = 78)**	**MF-DPI** **100 μg BID** **(n = 74)**	**BDP** **168 μg BID** **(n = 81)**
Age, mean y	8.4	8.1	8.0
Sex, n			
Girls	35	26	32
Boys	43	48	49
Race, n			
White	66	61	64
Other	12	13	17
Weight, mean kg	34.1	33.2	32.3
Asthma duration, mean y	5.3	4.8	5.1
Theophylline use, n	1	2	0
Baseline % predicted FEV_1_, mean	85.2	86.1	87.4

BID = twice daily; BDP = beclomethasone dipropionate; FEV_1 _= forced expiratory volume in 1 second; MF-DPI = mometasone furoate administered via a dry powder inhaler; QD AM = once daily in the morning.

### Safety

The study drugs were both well-tolerated with similar AE rates reported between treatment groups (Table 2). The most frequently reported AE were upper respiratory tract infections, reported by 47% to 51% of patients. Most AEs were considered unrelated to study drug. The most frequently reported related AEs were headache (MF-DPI 200 μg QD AM, 8%; MF-DPI 100 μg BID, 4%; BDP-MDI 168 μg BID, 2%) and oral candidiasis (4% in each treatment group). The incidence of pharyngitis was also similar among treatment groups. No deaths were reported throughout the study. Few treatment interruptions or discontinuations due to AEs were observed. Five treated patients (MF-DPI 200 μg QD AM, n = 3; MF-DPI 100 μg BID, n = 1; and BDP 168 μg BID, n = 1) and 1 screening patient experienced SAEs, however, none of these events were considered to be related to treatment. Three patients discontinued the study and 1 patient interrupted treatment because of a SAE. Neither the number nor nature of the SAEs reported suggested a differential risk of SAEs among the treatment groups. AEs reported by ≥ 20% of the study population are shown in Table 2.

**Table 2:** Adverse Events in ≥ 20% of Study Population

**Adverse Event, n (%)**	**MF-DPI** **200 μg QD AM** **(n = 78)**	**MF-DPI** **100 μg BID** **(n = 74)**	**BDP** **168 μg BID** **(n = 81)**
Allergy	22 (28)	11 (15)	20 (25)
Fever	18 (23)	20 (27)	26 (32)
Headache	33 (42)	27 (36)	25 (31)
Viral infection	27 (35)	26 (35)	29 (36)
Nasal congestion	7 (9)	17 (23)	11 (14)
Pharyngitis	26 (33)	25 (34)	25 (31)
Rhinitis	29 (37)	20 (27)	22 (27)
Sinusitis	15 (19)	15 (20)	11 (14)
Upper respiratory tract infection	38 (49)	35 (47)	41 (51)

BID = twice daily; BDP = beclomethasone dipropionate; MF-DPI = mometasone furoate administered via a dry powder inhaler; QD AM = once daily in the morning.

**Figure 2:**
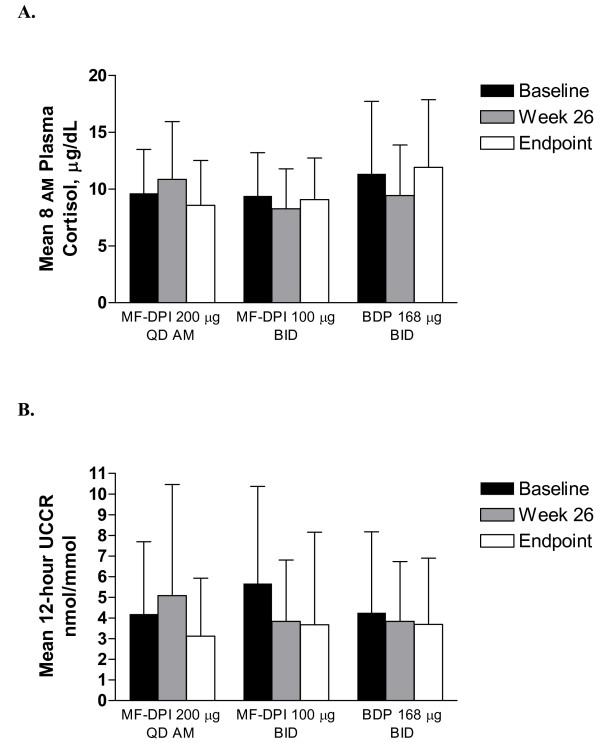
**(A) 8 AM plasma cortisol levels and (B) 12-hour urinary free cortisol levels adjusted for creatinine (UCCR) at baseline and endpoint**. Endpoint was defined as the measurement taken closest to the last day of study treatment. Bars indicate means + standard deviations. BID = twice daily; BDP = beclomethasone dipropionate; MF-DPI = mometasone furoate administered via a dry powder inhaler; QD AM = once daily in the morning.

The rate of clinical asthma exacerbations was similar among treatment groups, and there were no differences between treatment groups in the time to first asthma worsening event (*P *= 0.370). The median times to first asthma worsening event for the MF-DPI 200 μg QD AM, MF-DPI 100 μg BID, and BDP-MDI 168 μg treatment groups were 31, 35, and 17 weeks, respectively. Although patients who experienced asthma worsening were not necessarily discontinued from the study, 2 patients receiving MF-DPI 200 μg QD AM, 2 patients receiving MF-DPI 100 μg BID, and 3 patients receiving BDP 168 μg BID discontinued due to asthma worsening. No significant changes among treatment groups were observed in the mean 8 AM plasma cortisol levels from baseline even though the doses tested (ie, MF-DPI 200 μg QD AM and MF-DPI 100 μg BID), were twice as much as the approved doses (Figure 2A). The 12-hour urinary cortisol adjusted for creatinine (UCCR) was similar between treatment groups at endpoint, with mean values of 3.69 nmol/mmol for the BDP 168 μg BID group, 3.12 nmol/mmol for the MF-DPI 200 μg QD AM group, and 3.67 nmol/mmol for the MF-DPI 100 μg BID group (Figure 2B).

Plasma samples were collected for measuring MF-DPI concentrations from a total of 92 patients (MF-DPI 100 μg BID, n = 30; MF-DPI 200 μg QD AM, n = 34; BDP 168 μg BID, n = 28). At weeks 26 and 52, predose plasma MF-DPI concentrations were below the limit of quantitation in the majority of patients who were randomized to receive MF-DPI (MF-DPI 100 μg BID, 88% [week 26], 93% [week 52]; MF-DPI 200 μg QD AM, 78% [week 26], 93% [week 52]). As expected, no patient in the BDP 168 μg BID treatment group had a plasma MF-DPI concentration above the limit of quantitation.

## Discussion

This study evaluated the safety of MF-DPI at doses twice as high as the approved dose of 100 μg QD for children. BDP was chosen as the active comparator because it is an established corticosteroid used in treating asthma in children. The dose of BDP used during the clinical trial was the approved dose. A limitation of the study is that it evaluated the CFC formulation of BDP, which is no longer marketed. However, BDP-CFC is an established reference standard and its use as a comparator for adverse events and time to first exacerbation remains valid. Another limitation is the open-label design of the study, which may have introduced bias in regards to subjective outcomes such as patient reporting of adverse events. An open-label study design was employed so that the study could be amended or terminated if any treatment groups experienced unexpected trends in the incidence or severity of adverse events while the study was ongoing. Although inability to use MF-DPI device or peak flow meter was a basis for exclusion from this study, it must be emphasized that a majority of children are able to use the inhaler device correctly.

MF-DPI 200 μg QD AM and MF-DPI 100 μg BID dosing strategies were well tolerated and had similar AE profiles to BDP 168 μg BID. No clinically significant changes in laboratory parameters, vital signs, or physical examination findings over the course of the study in any treatment group were observed. Although the original study protocol called for the evaluation of HPA-axis effects with adrenocorticotropic hormone (ACTH)-stimulated cortisol testing, the protocol was amended such that 8 AM plasma cortisol and 12-hour UCCR replaced ACTH stimulation for cortisol testing. High baseline urinary cortisol levels were observed and notable variations in 12-hour UCCR levels were evident between patients, which accounted for the relatively large standard deviations for mean 12-hour UCCR values. This variability may have been due to the inherent shortcomings associated with a 12-hour urine collection period versus a more accurate 24-hour urine collection period. Overall, urine volumes tended to be lower at follow-up visits than at baseline and in some cases showed extreme change from the baseline volume, which suggests that urine collections may have been incomplete in the home environment. No significant differences between treatments were observed for 8 AM plasma cortisol levels at weeks 26, 52, and endpoint. These results are consistent with previous studies of ICS therapies in pediatric populations, [9-13] although acute adrenal crisis has been previously reported in children receiving high doses of several ICSs, including beclomethasone dipropionate and budesonide, but primarily fluticasone propionate.[14]

The results of the current study are similar to those from another clinical trial designed to evaluate the HPA-axis effects of 29 days of treatment with MF-DPI 100 μg BID, 200 μg BID, 400 μg BID, or placebo in children ages 6–11 years.[15] The MF-DPI 100 μg BID and 200 μg BID doses had no statistically significant effects on the 12-hour plasma cortisol area under the curve (AUC) compared with placebo. However, the MF-DPI 400 μg BID dose (8 times the approved dosage) significantly decreased the 12-hour AUC (*P *= 0.05). At day 29, cosyntropin stimulation responses were not significantly different between any of the treatment groups. No clinical AEs related to adrenal function were seen in the MF-DPI 400 μg BID group or any other, which indicates a wide HPA-axis safety margin for MF-DPI in children.

In a separate 52-week safety study of the impact of MF-DPI 100 μg BID, MF-DPI 100 μg QD AM, or MF-DPI 200 μg QD AM versus placebo on growth velocity and the HPA axis in 187 children (aged 4–9 y) with mild persistent asthma, MF-DPI did not show an effect on the HPA axis.[16] At the end of the treatment period, mean plasma cortisol values were highest in the 100-μg QD AM group, followed (in descending order) by the 100 μg BID, 200 μg QD AM, and placebo groups.

The effect of mometasone furoate on the HPA axis in children has also been studied using a metered dose inhaler formulation (MF-MDI). In a 52-week trial, 205 steroid-naive children (aged 4–9 y) with mild persistent asthma received MF-MDI 100 μg QD PM, MF-MDI 100 μg BID, or placebo.[17] Cortisol testing showed no HPA-axis effects with MF-MDI at any dose.

## Conclusion

These findings suggest that long-term treatment with MF-DPI 200 μg QD AM or MF-DPI 100 μg BID is safe in pediatric patients with persistent asthma who require ICS therapy, with no significant effects on HPA-axis function over at least 1 year. The doses tested here were twice as high as the 100 μg QD dose approved for children 4–11 years old. The AEs associated with MF-DPI use in children were similar to those observed when children are treated with BDP.

## Competing interests

Dr. Noonan has served as an Advisory Board participant for Schering-Plough and an Advisory Board and Speaker's Bureau participant for AstraZeneca. He has been the recipient of research grants from AstraZeneca, Dey Laboratories, Genentech, GlaxoSmithKline, Novartis, Amgen, Sepracor and Schering-Plough.

Dr. Leflein has served as a consultant with AstraZeneca, Aventis, Merck and Pfizer. He has participated in Speaker's Bureaus for AstraZeneca, Pfizer, Merck and Schering-Plough. In addition, he has received research grants from AstraZeneca, Merck, Novartis and DYAX.

Dr. Corren has served as a consultant with AstraZeneca, GlaxoSmithKline, Schering-Plough and Amgen. He has participated in Speaker's Bureaus for AstraZeneca, GlaxoSmithKline, Schering-Plough and Sepracor. He has received grants for research from AstraZeneca, GlaxoSmithKline, Schering-Plough, Amgen, Sepracor, Abbott, and SkyePharma.

Dr. Staudinger is an employee of Schering-Plough.

## Authors' contributions

MN, JL, and JC were involved in the collection of clinical data and interpretation of the results. HS was involved in the design of the study and interpretation of the results. All authors critically revised the manuscript, and have read and approved the final version.

## Appendix A

List of study sites and principal investigators:

Allergy, Immunology & Asthma Medical Group, Inc., Stockton, CA (George Bensch, MD); Asthma and Allergy Clinical Research Center, New Brunswick, NJ (William R. Bernstein, MD); National Jewish Medical and Research Center, Denver, CO (Mark Boguniewicz, MD); San Diego, CA (Gary A. Cohen, MD); Conyers, GA (Robert M. Cohen, MD); Allergy Research Foundation, Inc., Los Angeles, CA (Jonathan Corren, MD); The Asthma Center, Forked River, NJ (Donald Dvorin, MD); Clinical Research Institute, Minneapolis, MN and Allergy & Asthma Specialists, Minneapolis, MN (Harold B. Kaiser, MD); Clinical Research Institute of Southern Oregon, LLC, Medford, OR and Clinical Research Institute, Grants Pass, OR, and Clinical Research Institute, Klamath Falls, OR (Edward M. Kerwin, MD); Children's Research Institute at All Children's Hospital, St. Petersburg, FL (Jeffrey Ewig, MD); Ypsilanti, MI (Jeffrey Leflein, MD); Breath of Life Research Institute, Houston, TX (Stephen Miles, MD); ICSL-Clinical Studies, Normal, IL (Anjuli S. Nayak, MD); Allergy Associates Research Center, Portland, OR (Michael Noonan, MD); Colorado Allergy and Asthma Centers, PC, Aurora, CO (David Pearlman, MD); Allergy Associates Medical Group Inc., San Diego, CA (Bruce M. Prenner, MD); Ct. Asthma and Allergy Center, LLC, West Hartford, CT (James P. Rosen, MD); Tucson, AZ (Leonard Schultz, MD); Fort Collins, CO and Loveland, CO (Janet Seeley, MD); ASTHMA, Inc., Seattle, WA (Gail Shapiro, MD); Brooklyn, NY (Bernard Silverman, MD); Allergy and Asthma Care of Florida, Ocala, FL (G. Edward Stewart II, MD); Kirkland, WA and Bellevue, WA, and Seattle, WA (D. Robert Webb, MD); Toledo Center for Clinical Research, Sylvania, OH (John A. Winder, MD).

## Pre-publication history

The pre-publication history for this paper can be accessed here:


